# Long-term Results of Conversion Therapy for Initially Unresectable Gastric Cancer: Analysis of 122 Patients at the National Cancer Center in China

**DOI:** 10.7150/jca.35527

**Published:** 2019-10-15

**Authors:** Tongbo Wang, Nianchang Wang, Hu Ren, Hong Zhou, Aiping Zhou, Jing Jin, Yingtai Chen, Dongbing Zhao

**Affiliations:** 1 Department of Pancreatic and Gastric Surgical Oncology, National Cancer Center/National Clinical Research Center for Cancer/Cancer Hospital, Chinese Academy of Medical Sciences and Peking Union Medical College, Beijing 100021, China; 2 Department of Medical Oncology, National Cancer Center/National Clinical Research Center for Cancer/Cancer Hospital, Chinese Academy of Medical Sciences and Peking Union Medical College, Beijing 100021, China; 3 Department of Radiation Oncology, National Cancer Center/National Clinical Research Center for Cancer/Cancer Hospital, Chinese Academy of Medical Sciences and Peking Union Medical College, Beijing 100021, China

**Keywords:** Gastric cancer, Conversion therapy, Adjuvant chemotherapy, Unrespectable gastric cancer, Conversion surgery, Chemotherapy

## Abstract

**Purpose:** To assess the long-term survival and prognostic factors of conversion therapy in patients with initially unresectable gastric cancer.

**Patients and methods:** We conducted a retrospective study of clinicopathological and survival data of 122 consecutive patients who were diagnosed with initially unresectable gastric cancer and underwent the conversion surgery after systemic chemotherapy at the China National Cancer Center between May 2006 and May 2017.

**Results:** For all the 122 patients, the 3- and 5-year overall survival (OS) rates from the date of chemotherapy initiation were 61.0% and 52.0%, respectively, with a median OS of 63.6 months. During follow-up, the recurrence was observed in 49 (40.1%) patients who underwent conversion surgery. According to the multivariate COX regression analysis, receipt of postoperative adjuvant chemotherapy (POAC) was the only significant independent predictor of a favorable OS (HR 0.40; 95% CI 0.18-0.85, *P*=0.017). Log-rank analysis showed that POAC group experienced a survival advantage in terms of PFS when compared with observation group (HR 0.53, 95%CI 0.31-0.92, *P*=0.009).

**Conclusions:** Conversion therapy may provide long-term survival for patients with initially unresectable gastric cancer. Postoperative adjuvant chemotherapy might be recommended for patients who underwent conversion therapy.

## Introduction

Gastric cancer is the fifth most prevalent cancer and the third leading cause of cancer death worldwide^1^. Surgical resection is the major curative treatment for gastric cancer, but approximately two-thirds of patients have unresectable disease at the time of diagnosis such as local invasion, peritoneal dissemination, liver metastasis, or extra-regional lymph node metastasis^2^. In spite of recent developments in chemotherapy, the overall survival time of patients with unresectable gastric cancer remains to be around 12-14 months^3^-^6^.

Conversion surgery, or the notion that surgical treatment aimed at R0 resection following systemic chemotherapy for tumors initially considered unresectable of marginally resectable for technical and/or oncological reasons^7^, is one of the new therapeutic approaches for unresectable gastric cancer and successful treatment results have been reported^8^-^11^.However, the sample sizes of the previous studies are relatively small and the following issues remain to be clarified: the indications for the operation, the best chemotherapy regimen, and the best timing of the operation. Moreover, there is no evidence for whether postoperative adjuvant chemotherapy is necessary and what factors can predict the prognosis of these patients.

As such, we performed a retrospective analysis to evaluate the outcomes of curative intent conversion surgery following chemotherapy in patients with initially unresectable gastric cancer in terms of progress-free survival (PFS), overall survival (OS), recurrence and prognostic factors. We also examined the patients' survival according to different preoperative chemotherapy regimens to aid in the selection of optimal adjuvant treatment for patients with initially unresectable gastric cancer. To the best of our knowledge, this cohort is the largest to date in the conversion surgery for patients with unresectable gastric cancer.

## Materials and Methods

### Selection of patients

We conducted a retrospective selection of patients who were diagnosed gastric cancer and received systemic chemotherapy followed by surgery at the China National Cancer Center between May 2006 and May 2017. The inclusion criteria included: (1) histologically confirmed gastric adenocarcinoma by upper gastrointestinal endoscopy prior to treatment; (2) unresectable features detected by thoracic, abdominal and pelvic multidetector computed tomography (MDCT) or magnetic resonance imaging (MRI) for liver metastasis or fluorodeoxyglucose positron emission tomography (FDG-PET) before initial treatment; (3) patients that underwent systemic chemotherapy prior to surgery; (4) patients that underwent curative intent resection of primary lesion plus metastasectomy if needed. The exclusion criteria included: (1) patients that underwent neoadjuvant chemotherapy prior to surgery; (2) patients that underwent palliative resection or bypass operation; (3) incomplete clinical or pathological data registered. Patients with initially unresectable gastric cancer were considered if they had one or more of the following factors: tumor invasion to adjacent organ, liver metastasis, peritoneal metastasis (P+), extra-regional lymph nodes metastasis (ERLN+) such as para-aortic LN, or other distant metastasis. In addition, all patients included had an Eastern Cooperative Oncology Group performance status (PS) of 0-1. All study procedures were approved by the Institutional Review Board at the China National Cancer Center.

### Chemotherapy regimens and response evaluation

All patients received systemic chemotherapy at Department of Internal Medicine of China National Cancer Center. The chemotherapy regimen was decided by medical oncologists with a pretreatment evaluation consisted of physical examination, complete blood count, hepatic function, serum tumor marker assessment and electrocardiogram.

The tumor response was classified according to the *Response Evaluation Criteria in Solid Tumors* (RECISTversion 1.1) 12 and the adverse events of chemotherapy were assessed according to the *National Cancer Institute-Common Terminology Criteria for Adverse Events* (NCI-CTCAE version 4.0).

### Conversion surgery

All the patients underwent gastrectomy and standardized D2 or more lymph node dissection, plus metastasectomy if needed, with curative intent. The extent of gastrectomy, totally or sub-totally depended on the site and size of the primary tumor. If the CT and/or FDG-PET scan revealed complete remission of metastatic lesion after systemic chemotherapy and there was no macroscopic metastatic lesion at exploratory laparotomy, gastrectomy with D2 lymph node dissection was regarded as R0 resection. To ascertain the safety of conversion surgery, we evaluated the operative time, blood loss, operative mortality, and surgical morbidity.

### Histopathological examination

All the resected specimens were histopathologically analyzed to evaluate the extent of residual disease, pathological response to chemotherapy, and final pathological tumor stage. The tumor staging was determined according to *the 7^th^ American Joint Committee on Cancer (AJCC) TNM Staging Classification for Carcinoma of the Stomach*^13^.

The histological grade of tumor regression was classified based on the Mandard tumor regression grade (TRG)^14^. TRG 1 (complete regression) shows absence of residual cancer and fibrosis extending through the different layers of the esophageal wall; TRG 2 is characterized by the presence of rare residual cancer cells scattered through the fibrosis; TRG 3 features an increase in the number of residual cancer cells, but fibrosis still predominated; TRG 4 shows residual cancer outgrowing fibrosis; and TRG 5 is characterized by absence of regressive changes.

### Postoperative adjuvant chemotherapy and follow-up

Postoperative adjuvant chemotherapy (POAC) was recommended for all the patients who underwent conversion surgery. However, some patients declined postoperative chemotherapy due to various reasons. The chemotherapy regimens of POAC were based on the clinical response, pathological tumor grade and tumor regression grade of the preoperative chemotherapy.

All the patients were followed up every 3 months for the first 3 years and every 6 months afterwards until 5 years post-surgery. The follow-up content included physical examination, complete blood count, hepatic function, serum tumor marker assessment, thoracic, abdominal, and pelvic CT scanning and/or gastrointestinal endoscopy.

### Statistics

Continuous variables were expressed as median value and range or interquartile range (IQR). The OS was calculated from the date of chemotherapy initiation to the date of death or the last follow-up. The PFS was calculated from the date of chemotherapy initiation to the date of detection of recurrence or progression (R1/R2 resection) or death of any cause.The Kaplan-Meier method was used to analyze the survival data, the log-rank test and multivariate COX regression to compare the survival rates.

The statistical analysis was performed with SPSS v25 (IBM Corp., Chicago, IL, USA), Stata v14 (StataCorp, College Station, TX, USA) and Graphpad Prism 7 (GraphPad Software, San Diego, California, USA).

## Results

**Figure 1:**
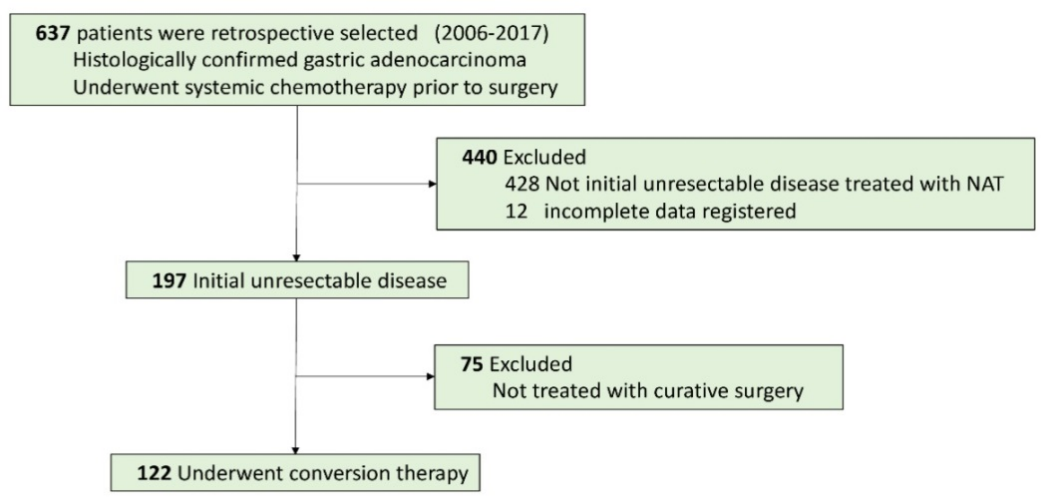
Patient Flowchart.

**Figure 2:**
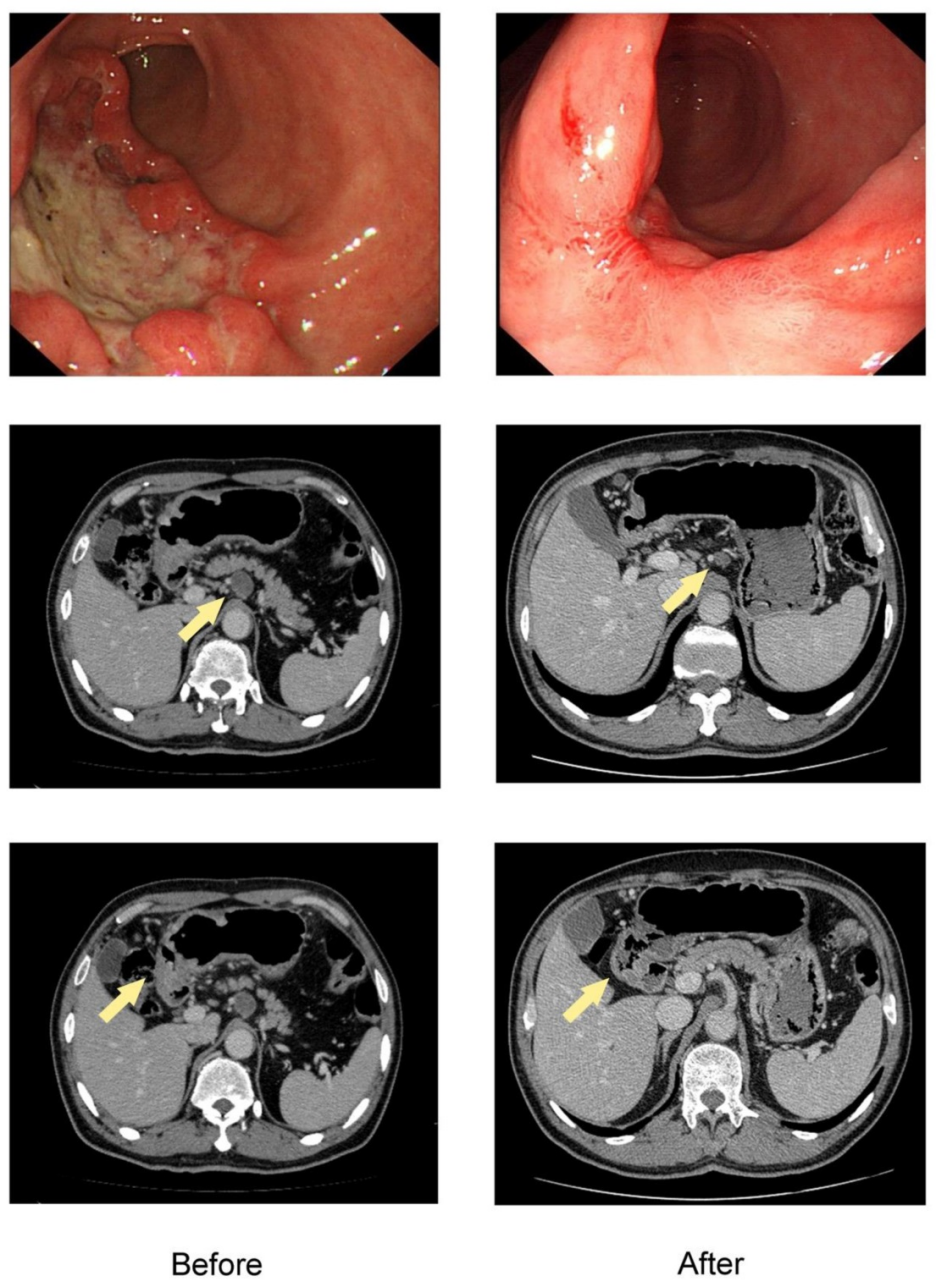
The endoscopic and CT images of the representative patient treated with conversion therapy. Baseline (left) and after 4 cycles of SOX chemotherapy (right) of the primary tumor and ERLN. The lesion of this patient was initially defined as unresectable due to ERLN+. The shrinkage was obtained both in the primary tumor and ERLN and the response was considered to be a PR, which was confirmed by the RECIST criteria.

A total of 637 patients diagnosed with gastric adenocarcinoma were retrospective selected in China National Cancer Center database. Of these, 122 patients with initially unresectable gastric cancer treated with systemic chemotherapy followed by curative intent resection met all the inclusion criteria (Figure 1). The median age was 56 years (range 28-78), and there were 88 males (72.1%). The clinicopathological characteristics at initiation of treatment were shown in Table 1. The endoscopic and CT images of the representative patient treated with conversion therapy were shown in Figure 2.

**Figure 3:**
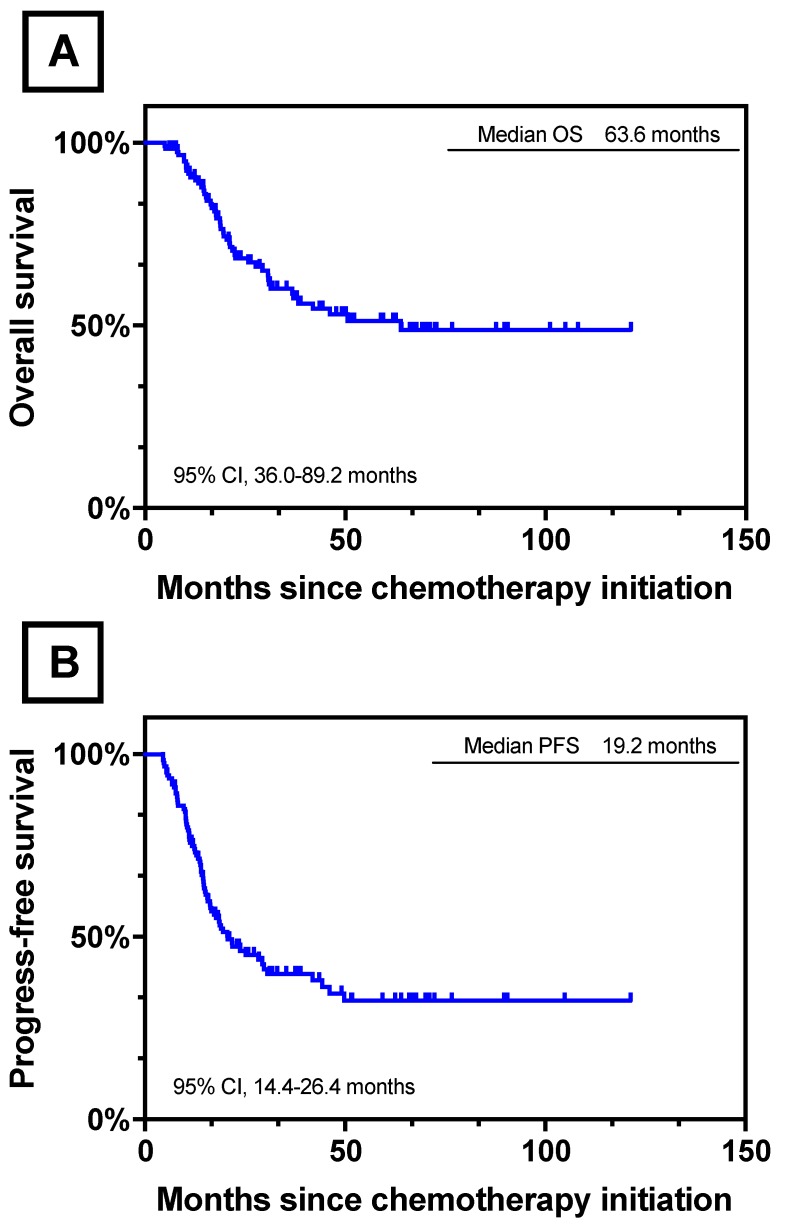
Survival Curves for 122 Patients who underwent conversion surgery. (A) OS for 122 Patients who underwent conversion surgery.; (B) PFS for 122 Patients who underwent conversion surgery.

After a median follow-up of 63.6 months (range 4.9-121.3 months), the respective 3- and 5-year OS rate of all the 122 patients were 61.0% and 52%, with a median OS of 63.6 months (95% CI, 36.0-89.2 months). The 3- and 5- year PFS rates were 34.0% and 26.0%, respectively. The median PFS was 19.2 months (95% CI, 14.4-26.4 months). The Kaplan-Meier curves of OS and PFS of 122 patients were shown in Figure 3.

### Unresectable factors

**Figure 4:**
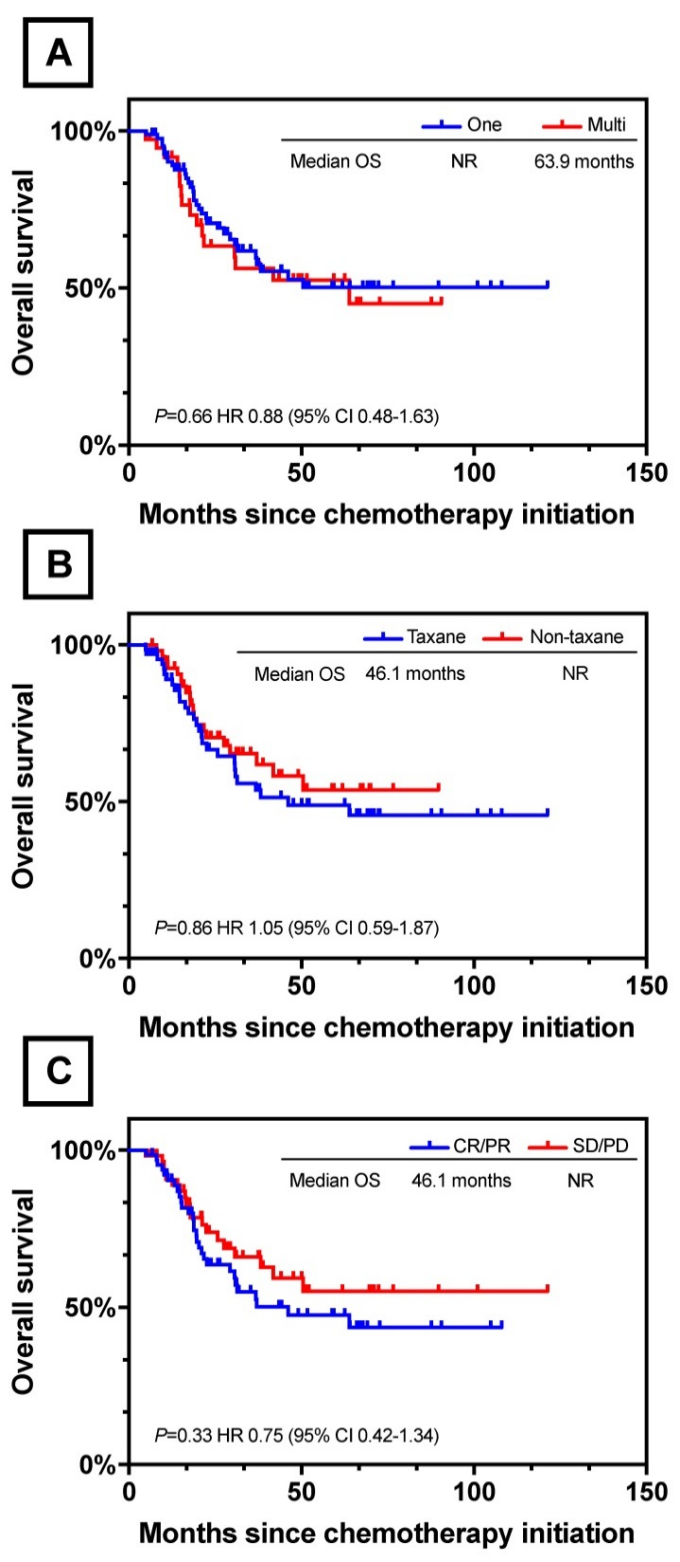
Survival Curves for Different Clinical Characteristic. (A) OS between one and multi unresectable factors. (B) OS between different chemotherapy regimens. (C) OS between clinical response and non-response.

Of the 122 patients, 85 (69.7%) were identified to have one unresectable factor each: tumor invasion to adjacent organ in 13 patients, ERLN+ in 55 (para-aortic LN, n=49; Virchow's LN, n=8; pelvic LN, n=1), liver metastasis in 12, P+ in 3, and other distantmetastasis in 2 (including 1 with lung metastasis and 1 with Krukenberg tumor); while 27 (30.3%) were identified to have multiple unresectable factors each: two unresectable factors in 35 patients (T4b-ERLN+, n=17; liver-ERLN+, n=4; T4b-P+, n=4; T4b-liver, n=3; P+-ERLN+, n=2; ovary-ERLN+, ovary-T4b, ovary-P+, lung-liver, lung-ERLN+, n=1); 2 patients presented three unresectable factors of 1 T4b-P+-ERLN+ and 1 T4b-liver-ERLN+. There was no significant difference in the OS rate between the patients with one and multiple unresectable factors (one vs multi; NR vs 63.9 months; *P*=0.66, Figure 4A).

### Chemotherapy and clinical response

Chemotherapy regimens and clinical effects are summarized in Table 2. The chemotherapy regimens were classified into two groups. For 67 patients in the taxane based group, chemotherapeutic schemes included paclitaxel-S-1 (n=39), paclitaxel-oxaliplatin (n=7) and docetaxel-cisplatin-S-1 (n=16). For 55 patients treated with non-taxane based therapy included S-1-oxaliplatin (n=41), S-1-cisplatin (n=10) and 5-fu-cispaltin (n=4). The median number of cycles administrated was 4 in both groups (IQR; taxane based 2-18, non-taxane based 1-15). With respect to toxicity, 20.5% (25/122) of all the patients had grade 3 or 4 adverse events. Bone marrow hypocellular was the most frequent toxicity in both two groups with 11 in taxane based and 10 in non-taxane based. There was no treatment-related death in the duration of systemic chemotherapy. There was no statistically difference between taxane based and non-taxane based groups in median OS (taxane vs non-taxane; 46.1 months vs NR; *P*=0.86, Figure 4B).

Of the 122 patients, 1 patient had a complete response (CR), 64 had a partial response (PR), and 55 had stable disease (SD). In the taxane group, 34 patients achieved CR/PR and the overall response rate was 50.7%. For patients in non-taxane based group, 31 patients had a CR/PR and the overall response rate was 56.4%. There was no significant difference in clinical response between these two groups (*P*=0.536). We further divided the whole 122 patient cohort into two groups according to the response to preoperative chemotherapy: the response group (CR/PR, n=65) and the non-response group (SD/PD, n=57). The Kaplan-Meier and log-rank analysis showed no statistically difference between response group and non-response group in median OS (response vs non-response; 46.1months vs NR; *P*=0.33, Figure 4C).

### Surgery and pathological findings

Table 3 summarized the surgical and pathological findings of the patients who underwent conversion surgery. In all, 26 patients (21.3%) underwent proximal gastrectomy, 61 (50.0%) of distal gastrectomy and 35 (28.7%) of total gastrectomy. D2 lymph node dissection were performed in all the patients and extended para-aortic lymph node dissection (PAND) if they were para-aortic lymph node -positive (cN3) at presentation. The median blood loss was 200ml (IQR 100-200) and the median surgery duration was 190min (IQR 160-240), respectively. Of the 122 patients, 113 (92.6%) achieved R0 resections, while for the remaining 9 patients, there were R1 resection for microscopic margin positive (n =5, 4.1%), R2 resection for peritoneal dissemination (n=1, 0.8%), unresectable liver metastasis (n=2, 1.6%) and lung metastasis (n=1, 0.8%). Mortality or serious complications were not observed.

According to the Mandard Tumor Regression Grade (TRG), the pathological response rate of the primary tumors was 50.9%, which included TRG 1-3 in 62 patients. The pathological response was classified as TRG 1 (complete regression) in the primary tumor of 9 patients, 2 of whom were observed with recurrence during the follow-up period.

### Postoperative chemotherapy and recurrence

Among the 122 patients, 80 (65.6%) received postoperative adjuvant chemotherapy after conversion surgery. The most frequently used chemotherapy regimens were S-1 alone (n=36) and S-1 plus platinum (n=28) and treatment was given for median cycles of 3 (IQR 2-6).

During the follow-up, recurrence was observed in 49 (40.1%) patients who underwent conversion surgery. The recurrence site included the abdominal LNs (n=21), peritoneum (n=10), liver (n=12) and other organs (ovary, n=2; lung, n=1; kidney, n=1; bone, n=1). In 29 (59.2%) of these patients with recurrence, the type of recurrence was mostly consistent with their initial metastatic disease.

### Univariate and multivariate survival analysis in OS

Table 4 showed the results of univariate and multivariate analysis concerning OS. In the univariate analysis, the clinical T4a (HR 7.91; 95% CI, 1.78-35.12; *P=*0.0065), ypN3a (HR 6.19; 95% CI, 2.39-16.02l; *P=*0.0002), ypN3b (HR 10.28; 95% CI, 3.85-27.43; *P<*0.0001), and lymphatic recurrence (HR, 2.64; 95% CI, 1.33-5.27; *P=*0.0058) were associated with OS. Age, sex, tumor location, clinical N stage, unresectable factor, residual tumor, ypT stage, pathological response, clinical response, chemotherapy regimens, no. of chemotherapy cycles, and no. of postoperative chemotherapy cycles were not significantly associated with OS.

According to the multivariate COX regression analysis, receipt of postoperativeadjuvant chemotherapy (HR 0.40; 95% CI, 0.18-0.85,*P*=0.017) was the only significant independent predictor of favorable OS, and while the types of unresectable factors, number of unresectable factors, chemotherapy regimens, R0 resection, clinical response and pathological response were not significantly associated with survival.

### Survival by POAC and observation groups

**Figure 5:**
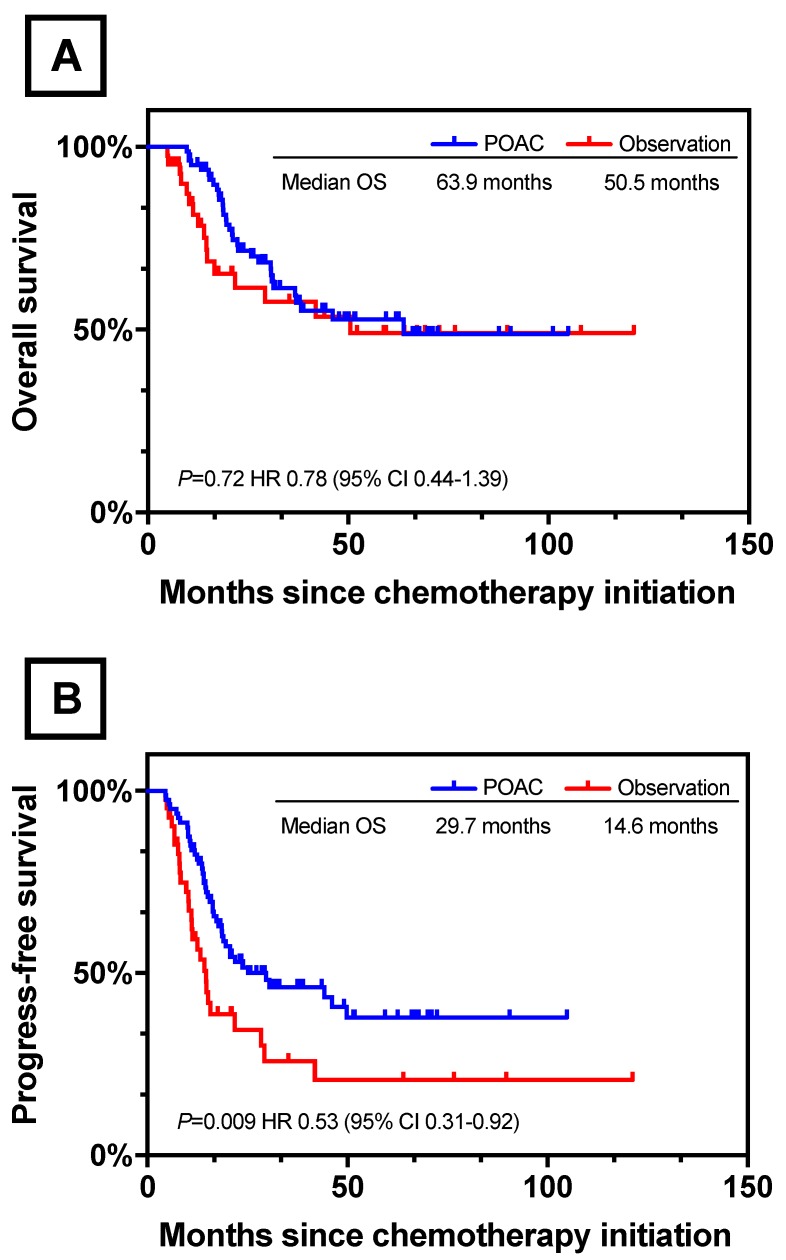
Survival Curves between POAC and observation. (A) OS Between POAC and Observation (B) PFS Between POAC and Observation.

Further survival analysis was performed between patients who had treated with postoperative chemotherapy (n=80) and observation (n=42). Patients' baseline characteristics were described in Table S1 and the two groups were well balanced. With respect to OS, no significant difference was found between POAC group and observation group (POAC 63.9 months vs observation 50.5 months, *P*=0.72, Figure 5A). However, POAC group experienced a survival advantage in terms of PFS when compared with observation group (POAC 29.7 months vs observation 14.6 months, P=0.009, Figure 5B).

## Discussion

There is scarce evidence of the necessity of postoperative chemotherapy for patients who underwent conversion surgery. A few studies have investigated the prognostic role of postoperative chemotherapy in the patients treated with conversion surgery. Fukuchi et al. reported that postoperative chemotherapy was not significantly associated with OS among the 40 patients treated with conversion surgery^15^. However, the limited sample size made it difficult to adequately adjust for all potential confounding factors. On the other hand, Satoh and his group suggested that postoperative chemotherapy with S-1 alone might be effective in treating stage IV gastric cancer if the latent tumor burden is minimal after R0 resection^9^. However, the conclusion was speculative due to a lack of survival analysis according to the receipt of postoperative chemotherapy. Our data clearly show that receipt of postoperative chemotherapy is a significant independent predictor of favorable OS. Besides, the PFS of POAC group was significantly better than the observation group (*P*=0.009). The results supported the current hypothesis that postoperative adjuvant chemotherapy with cautious follow-up should be accompanied with patients treated with conversion surgery^10^. While the mechanism underlying this phenomenon remained unclear, a possible explanation was that even though treated with systemic chemotherapy and curative surgery, there were still 49 (40.1%) patients had recurrence in the present study.

Sato et al.^16^ reported a median OS of patients who underwent conversion therapy of 47.8 months with 1-, 3-, and 5-year OS rates of 97.0, 63.6, and 42.4%, respectively, in a multi-institute analysis implemented in 2017 in 100 patients with unresectable gastric cancer. The R0 resection rate was 84.8% (28/33) and R0 resection led to significantly longer OS than non-R0 resection (*P*=0.0002). Han et al. 10 reported that R0 resection was achieved after systemic chemotherapy in 26 (76.5%) of 34 patients with M1 gastric cancer, with a median OS from the time of surgery for R0 resection and R1-2 resection of 22.9 months and 7.8 months, respectively. The OS rate was statistically higher for patients with R0 resection than that for those with R1-2 resection (*P*=0.033). Fukuchi and his team^15^ reported a median OS of 53 months among the 40 patients who underwent conversion surgery with a 5-year OS rate of 43% and R0 resection rate in their study was 80.0%. Patients who underwent R0 resection had significantly longer OS duration than those who underwent R1 and R2 resection (*P*=0.03). Similarly, Saito et al.^17^ reported that R0 resection was a significant independent predictor of survival for stage IV gastric cancer patients treated with induction chemotherapy using S-1 and cisplatin followed by curative resection. The median OS in the present study was 63.6 months that was longer than those in the previous reports 10,15,16. The 3- and 5-year OS rates were 61.0% and 52.0%, respectively. This could be due to that the R0 resection rate in our cohort was higher than those of the previous studies (92.6% in the present cohort vs 84.8% in Sato's report and 76.5% in Han's report and 80.0% in Fukuchi's report). However, we found that there was no significant difference in the OS rate between the patients who underwent R0 resection and non-R0 resection (*P*=0.26) in the present study. This result was not consistent with those in the previous studies. A possible explanation of this phenomenon was that the number of cases in the non-R0 resection group was small due to the high R0 resection rate in the present study, therefore chance cannot be ruled out for this result.

Although conversion therapy has been attracting increasing attention, the eligible patients and definition of “unresectable tumor” or “non-curative status” for this concept remain unclear^18^. Saito et al.^17^ reported that negative para-aortic lymph nodes was an independent prognostic factor of survival for patients treated with induction chemotherapy followed by curative resection (*P*=0.002). On the other hand, Fukuchi and his team^15^ reported that patients with unresectable gastric cancer initially exhibiting one non-curative factor might obtain a survival benefit from conversion therapy (*P*=0.02). However, the survival rates did not differ significantly between the patients with one and multiple unresectable factors (*P*=0.66) in the present study. Moreover, the survival rates did not differ significantly between the patients with different unresectable factors such as clinical T4b, extra-regional lymph nodes metastasis, H1 and P1. Yoshida et al.^7^,^19^,^20^ proposed a new categories of classification for stage IV gastric cancer concerning conversion therapy. In this classification, stage IV gastric cancer was classified into four categories based on the absence (category 1 and 2) or presence (category 3 and 4) of macroscopically detectable peritoneal dissemination, which has a different biological outcome compared to hematological metastasis. Such classification of these categories can play an important role in designing future prospective cohort studies and/or randomized control trials to elucidate who may benefit from conversion therapy^21^. To the best of our knowledge, there are few randomized studies focusing on this clinical issue.

Sato et al.^16^ reported that the pathological response was a significant independent predictor of OS for patients who underwent conversion therapy. On the other hand, although evidence shows that pathological response is of demonstrated value in predicting the survival of gastric cancer patients who have received neoadjuvant chemotherapy, it is not comparable to ypN status or microscopic lymphovascular invasion as a prognostic predictor 22,23. Accordingly, the present study found that pathological response did not impact OS, but ypN3a (*P*=0.0002) and ypN3b (*P*<0.0001) were significantly associated with worse OS in the univariate analysis.

Fluoropyrimidine plus platinum and taxane based combination chemotherapy regimenswere recommended as first-line chemotherapy regimen for unresectable gastric cancer according to the NCCN guidelines. A recent study concerning conversion therapy reported that the median OS and patients converted to conversion surgery did not differ significantly between S-1 plus cisplatin group and S-1 plus paclitaxel group^15^. Similar results were also reported by Fukuchi et al 24 that response, severe toxicity and conversion surgery were not significantly different between S-1 plus cisplatin and S-1 plus oxaliplatin. Additionally, it has been reported 21,25 that docetaxel, cisplatin and S-1 (DCS) showed a overall response rate of 73.7% to 81% and conversion surgery rate of 33% to 59.6%, which seems to be promising for conversion therapy. However, the grade 3 or 4 toxicity was also in high frequency in DCS therapy 16. In the present study, no significant differences were found between taxane based and non-taxane based chemotherapy groups in terms of overall response rate, no. of courses and severe toxicities. Besides, chemotherapy regimens were not significantly associated with OS in either univatiate or multivariate analysis. Taken together, chemotherapy regimens might not associate the survival of the patients treated with conversion therapy and the selection of chemotherapeutic scheme should balance the effectiveness and toxicity.

It was believed that the degree of toxicity of preoperative chemotherapy is a critical problem because of potential adverse effects in operative morbidity and mortality. However, only 24 (19.7%) patients had grade 3 or 4 toxicity and all treatment-related toxicities were resolved with appropriate care in the present study. Moreover, treatment-related death or serious complications were not observed. In a word, these findings suggested that conversion therapy be safe and feasible.

Strengths and limitations should be considered when the study results are interpreted. In this study, we reviewed 122 consecutive patients with unresectable gastric cancer who underwent conversion surgery, which, to our knowledge, is the largest study population of this nature. Our study, for the first time, reported that receipt of postoperative chemotherapy had a favorable effect on the prognosis of patients with unresectable gastric cancer treated with conversion therapy. However, this retrospective study was limited by its exploratory nature, and no control group was available for comparison. Moreover, even in the patients with good response to induction chemotherapy, residual cancer had to be histologically confirmed in the most cases. Unfortunately, our study failed to record this information. Finally, this was a single-institutional analysis with significant treatment heterogeneity.

## Conclusions

The present study showed that conversion therapy may provide long-term survival for patients with initially unresectable gastric cancer. Postoperative chemotherapy might be recommended for patients who underwent conversion therapy. Large-scale, multicenter randomized trials are needed to further verify the findings in our study.

## Supplementary Material

Supplementary tables.

## Figures and Tables

**Table 1:** Clinicopathological characteristics of 122 patients who underwent conversion surgery.

Characteristic		No. (%)
Age	median (range)	56 (28-78)
Sex		
	Male	88 (72.1)
	Female	34 (27.9)
Lauren Type		
	Intestinal	53 (43.4)
	Diffuse	48 (39.3)
	Mixed	21 (17.2)
Bormann Type		
	1	17 (13.9)
	2	29 (23.8)
	3	68 (55.7)
	4	8 (6.6)
Grade		
	Well differentiate	3 (2.5)
	Moderately differentiate	18 (14.8)
	Poor differentiate	101(82.8)
Tumor location		
	Upper	29 (23.8)
	Middle	39 (32)
	Lower	53 (43.4)
	Whole	1 (0.8)
Clinical T stage		
	T1+T2	17 (13.9 )
	T3	37 (30.3)
	T4a	32 (26.2)
	T4b	36 (29.5)
Clinical N stage		
	N0+N1	8 (6.6)
	N2	39 (32)
	N3	75 (61.5)
Unresectable factor		
	Tumor invasion to adjacent organ	13 (10.7)
	ERLN+	55 (45.1)
	Liver	12 (9.8)
	P+	3 (2.5)
	Other	2 (1.6)
	Multi-unresectable factors	37 (30.3)
Postoperative chemotherapy		
	Yes	80 (65.6)
	No	42 (34.4)
Recurrence		
	No	73 (59.8)
	Lymphatic	21 (17.2)
	Peritoneal	10 (8.2)
	Liver	12 (9.8)
	Other	6 (4.9)

**Table 2:** Chemotherapy regimens and clinical response.

		No. (%)		
		Taxane based	Non- taxane based	
Characteristic		( n = 67 )	( n = 55 )	*P* value
Overall response rate (%)		50.70%	56.40%	0.536
Clinical response				
	CR/PR	34	31	
	SD/PD	33	24	
No. of chemotherapy cycles				
	median (IQR)	4 ( 2-18)	4 ( 1-15)	0.912
Adverse events (grade 3 or 4)		13	12	0.82
	Vomiting	1	1	
	Bone marrow hypocellular	11	10	
	Hepatic failure	0	1	
	Paresthesia	1	0	

Abbreviation: CR, complete response; PR, partial response; SD, stable disease; PD, progressive disease; IQR: Interquartile Range.

**Table 3:** Surgical and pathological findings.

Characteristic		No. (%)
Operation procedure		
	Proximal gastrectomy	26 (21.3)
	Distal gastrectomy	61 (50.0)
	Total gastrectomy	35 (28.7)
Combined resection		
	Lung	1 (0.8)
	Liver	6 (4.9)
	Ovary	2 (1.6)
Operation time (min)		
	median (IQR)	190 (160-240)
		
Blood loss (ml)		
	median (IQR)	200 (100-200)
		
Residual tumor		
	R0	113(92.6)
	R1	5 (4.1)
	R2	4 (3.3)
ypT stage AJCC 7^th^		
	ypT0	5 (4.1)
	ypT1	8 (6.6)
	ypT2	16 (13.1)
	ypT3	46 (37.7)
	ypT4a	41 (33.6)
	ypT4b	6 (4.9)
ypN stage AJCC 7th		
	ypN0	38 (31.2)
	ypN1	17 (13.9)
	ypN2	23 (18.9)
	ypN3a	25 (20.5)
	ypN3b	19 (15.6)
Pathological response (Mandard grade)		
	TRG1	9 (7.4)
	TGR2	29 (23.8)
	TRG3	24 (19.7)
	TRG4	41 (33.6)
	TRG5	19 (15.6)

Abbreviation: AJCC 7^th^, the 7^th^ American Joint Committee on Cancer (AJCC) TNM Staging Classification for Carcinoma of the Stomach; TRG, tumor regression grade. IQR: Interquartile Range.

**Table 4:** Results of univariate and multivariate analyses of association between patient characteristics and OS.

		Univariable		Multivariable	
Variable		HR (95% CI)	*P* value	HR (95% CI)	*P* value
Age					
	≤65	Reference			
	>65	0.65(0.29-1.46)	0.3		
Sex					
	Male	Reference			
	Female	1.53(0.83-2.81)	0.17		
Tumor location					
	Upper	Reference			
	Middle	1.46(0.65-3.30)	0.36		
	Lower	1.77(0.82-3.84)	0.15		
	Whole	―			
Clinical T stage					
	T1+T2	Reference			
	T3	6.25(1.46-26.84)	0.014		
	**T4a**	**7.91(1.78-35.12)**	**0.0065**		
	T4b	3.93(0.87-17.82)	0.076		
Clinical N stage					
	N0+N1	Reference			
	N2	3.22(0.42-24.64)	0.26		
	N3	5.05(0.69-36.92)	0.11		
Unresectable factors					
	Tumor invasion to adjacent organ	Reference		Reference	
	ERLN+	1.91(0.58-6.33)	0.29	1.73(0.24-12.68)	0.59
	Liver	0.72(0.15-3.60)	0.69	1.11(0.09-14.20)	0.94
	P+	―	―	―	―
	Others	2.06(0.21-19.93)	0.53	116.92(2.09-6532.20)	0.02
	Multi-unresectable factors	1.70(0.50-5.85)	0.4	1.32(0.30-5.80)	0.72
Residual tumor					
	R0	Reference		Reference	
	R1/2	0.45(0.11-1.86)	0.27	0.58(0.09-4.00)	0.58
ypT stage AJCC 7th					
	ypT0	Reference			
	ypT1	0.65(0.09-4.65)	0.67		
	ypT2	0.52(0.07-3.73)	0.52		
	ypT3	1.80(0.42-7.66)	0.43		
	ypT4a	2.07(0.47-9.04)	0.33		
	ypT4b	2.66(0.49-14.60)	0.26		
ypN stage AJCC 7th					
	ypN0	Reference			
	ypN1	2.71(0.87-8.40)	0.085		
	ypN2	2.81(0.97-8.10)	0.056		
	**ypN3a**	**6.19(2.39-16.02)**	**0.0002**		
	**ypN3b**	**10.28(3.85-27.43)**	**<0.0001**		
Pathological response (Mandard grade)					
	TRG1	Reference		Reference	
	TRG2	1.50(0.41-5.46)	0.54	0.98(0.23-4.21)	0.98
	TRG3	0.97(0.24-3.90)	0.97	0.47(0.09-2.39)	0.36
	TRG4	2.20(0.65-7.47)	0.2	1.19(0.27-5.22)	0.82
	TRG5	2.44(0.67-8.90)	0.18	1.44(0.31-6.70)	0.64
Chemotherapy regimens					
	Non-taxane based	Reference		Reference	
	Taxane based	1.05(0.59-1.87)	0.86	1.34(0.70-2.55)	0.38
Clinical response					
	CR/PR	Reference		Reference	
	SD/PD	0.75(0.42-1.34)	0.33	0.77(0.36-1.63)	0.49
**Postoperative adjuvant chemotherapy**					
	No	Reference		Reference	
	**Yes**	0.78(0.44-1.39)	0.72	**0.40(0.18-0.85)**	**0.017**
Recurrence site					
	No	Reference			
	**Lymphatic**	**2.64(1.33-5.27)**	**0.0058**		
	Peritoneal	2.12(0.84-5.31)	0.11		
	Liver	1.74(0.73-4.14)	0.21		
	Other	1.03(0.24-4.42)	0.97		

Abbreviation: HR, hazard ratio; CI, confidence interval; P+, peritoneal metastasis; ERLN+, extra-regional lymph nodes metastasis; AJCC 7^th^, *the 7^th^ American Joint Committee on Cancer (AJCC) TNM Staging Classification for Carcinoma of the Stomach*; TRG, tumor regression grade; CR, complete response; PR, partial response; SD, stable disease; PD, progressive disease; IQR: Interquartile Range

## Abbreviations

PFS: progress-free survival
OS: overall survival
MDCT: multidetector computed tomography
MRI: magnetic resonance imaging
FDG-PET: fluorodeoxyglucose positron emission tomography
P+: Peritoneal metastasis
ERLN+: Extra-region lymph node metastasis
PS: Eastern Cooperative Oncology Group performance status
RECIST: Response Evaluation Criteria in Solid Tumors
NCI-CTCAE: National Cancer Institute-Common Terminology Criteria for Adverse Events
AJCC: American Joint Committee on Cancer
POAC: Postoperative adjuvant chemotherapy
TRG: tumor regression grade
IQR: Interquartile range
HR: Hazard ratio
CI: Confidence interval
LN: Lymph node
NR: Not reached
CR: complete response
PR: partial response
SD: stable disease
PD: progressive disease
PAND: Extended para-aortic lymph node dissection
DCS: docetaxel, cisplatin and S-1

## References

[1] Ferlay J, Soerjomataram I, Dikshit R, Eser S, Mathers C, Rebelo M (2015). Cancer incidence and mortality worldwide: sources, methods and major patterns in GLOBOCAN 2012. Int J Cancer.

[2] Vanhoefer U, Rougier P, Wilke H, Ducreux MP, Lacave AJ, Van Cutsem E (2000). Final results of a randomized phase III trial of sequential high-dose methotrexate, fluorouracil, and doxorubicin versus etoposide, leucovorin, and fluorouracil versus infusional fluorouracil and cisplatin in advanced gastric cancer: A trial of the European Organization for Research and Treatment of Cancer Gastrointestinal Tract Cancer Cooperative Group. J Clin Oncol.

[3] Yoshida K, Ninomiya M, Takakura N, Hirabayashi N, Takiyama W, Sato Y (2006). Phase II study of docetaxel and S-1 combination therapy for advanced or recurrent gastric cancer. Clin Cancer Res.

[4] Koizumi W, Narahara H, Hara T, Takagane A, Akiya T, Takagi M (2008). S-1 plus cisplatin versus S-1 alone for first-line treatment of advanced gastric cancer (SPIRITS trial): a phase III trial. Lancet Oncol.

[5] Bang YJ, Van Cutsem E, Feyereislova A, Chung HC, Shen L, Sawaki A (2010). Trastuzumab in combination with chemotherapy versus chemotherapy alone for treatment of HER2-positive advanced gastric or gastro-oesophageal junction cancer (ToGA): a phase 3, open-label, randomised controlled trial. Lancet.

[6] Koizumi W, Kim YH, Fujii M, Kim HK, Imamura H, Lee KH (2014). Addition of docetaxel to S-1 without platinum prolongs survival of patients with advanced gastric cancer: a randomized study (START). J Cancer Res Clin Oncol.

[7] Yoshida K, Yamaguchi K, Okumura N, Tanahashi T, Kodera Y (2016). Is conversion therapy possible in stage IV gastric cancer: the proposal of new biological categories of classification. Gastric Cancer.

[8] Suzuki T, Tanabe K, Taomoto J, Yamamoto H, Tokumoto N, Yoshida K (2010). Preliminary trial of adjuvant surgery for advanced gastric cancer. Oncol Lett.

[9] Satoh S, Okabe H, Teramukai S, Hasegawa S, Ozaki N, Ueda S (2012). Phase II trial of combined treatment consisting of preoperative S-1 plus cisplatin followed by gastrectomy and postoperative S-1 for stage IV gastric cancer. Gastric Cancer.

[10] Han DS, Suh YS, Kong SH, Lee HJ, Im SA, Bang YJ (2013). Outcomes of surgery aiming at curative resection in good responder to induction chemotherapy for gastric cancer with distant metastases. J Surg Oncol.

[11] Okabe H, Ueda S, Obama K, Hosogi H, Sakai Y (2009). Induction chemotherapy with S-1 plus cisplatin followed by surgery for treatment of gastric cancer with peritoneal dissemination. Ann Surg Oncol.

[12] Eisenhauer EA, Therasse P, Bogaerts J, Schwartz LH, Sargent D, Ford R (2009). New response evaluation criteria in solid tumours: revised RECIST guideline (version 1.1). Eur J Cancer.

[13] Washington K (2010). 7th edition of the AJCC cancer staging manual: stomach. Ann Surg Oncol.

[14] Mandard AM, Dalibard F, Mandard JC, Marnay J, Henry-Amar M, Petiot JF (1994). Pathologic assessment of tumor regression after preoperative chemoradiotherapy of esophageal carcinoma. Clinicopathologic correlations. Cancer-Am Cancer Soc.

[15] Fukuchi M, Ishiguro T, Ogata K, Suzuki O, Kumagai Y, Ishibashi K (2015). Prognostic Role of Conversion Surgery for Unresectable Gastric Cancer. Ann Surg Oncol.

[16] Sato Y, Ohnuma H, Nobuoka T, Hirakawa M, Sagawa T, Fujikawa K (2017). Conversion therapy for inoperable advanced gastric cancer patients by docetaxel, cisplatin, and S-1 (DCS) chemotherapy: a multi-institutional retrospective study. Gastric Cancer.

[17] Saito M, Kiyozaki H, Takata O, Suzuki K, Rikiyama T (2014). Treatment of stage IV gastric cancer with induction chemotherapy using S-1 and cisplatin followed by curative resection in selected patients. World J Surg Oncol.

[18] Terashima M (2016). Conversion therapy for gastric cancer: who can make conversion as successful as Goromaru?. Gastric Cancer.

[19] Yoshida K, Yamaguchi K, Okumura N, Osada S, Takahashi T, Tanaka Y (2011). The roles of surgical oncologists in the new era: minimally invasive surgery for early gastric cancer and adjuvant surgery for metastatic gastric cancer. Pathobiology.

[20] Yamaguchi K, Yoshida K, Tanaka Y, Matsuhashi N, Tanahashi T, Takahashi T (2016). Conversion therapy for stage IV gastric cancer-the present and future. Transl Gastroenterol Hepatol.

[21] Mieno H, Yamashita K, Hosoda K, Moriya H, Higuchi K, Azuma M (2017). Conversion surgery after combination chemotherapy of docetaxel, cisplatin and S-1 (DCS) for far-advanced gastric cancer. Surg Today.

[22] Mansour JC, Tang L, Shah M, Bentrem D, Klimstra DS, Gonen M (2007). Does graded histologic response after neoadjuvant chemotherapy predict survival for completely resected gastric cancer?. Ann Surg Oncol.

[23] Koh YW, Park YS, Ryu MH, Ryoo BY, Park HJ, Yook JH (2013). Postoperative nodal status and diffuse-type histology are independent prognostic factors in resectable advanced gastric carcinomas after preoperative chemotherapy. Am J Surg Pathol.

[24] Fukuchi M, Mochiki E, Ishiguro T, Ogura T, Sobajima J, Kumagai Y (2017). Efficacy of Conversion Surgery Following S-1 plus Cisplatin or Oxaliplatin Chemotherapy for Unresectable Gastric Cancer. Anticancer Res.

[25] Kinoshita J, Fushida S, Tsukada T, Oyama K, Okamoto K, Makino I (2015). Efficacy of conversion gastrectomy following docetaxel, cisplatin, and S-1 therapy in potentially resectable stage IV gastric cancer. Eur J Surg Oncol.

