# Supplementary material for: Long-term Results of Conversion Therapy for Initially Unresectable Gastric Cancer: Analysis of 122 Patients at the National Cancer Center in China
Source: J Cancer. 2019 Oct 15;10(24):5975–85. doi: 10.7150/jca.35527 (PMC6856572; doi:10.7150/jca.35527)
Supplement: Supplementary file 1 — Supplementary tables. [file jcav10p5975s1.pdf]

Table S1. Baseline characteristic between patients in POAC and observation group.

Characteristic		No. (%) Postoperative chemotherapy (n = 80)	Observation (n = 42)	*P* value
Age (median, range)		57 (30-75)	54 (28-78)	0.753
Male		60 (75.0)	28 (66.7%)	0.329
Histologic grade				0.113
	Well differentiate	2 (2.5)	1 (2.4)	
	Moderately differentiate	15 (18.8)	3 (7.1)	
	Poor differentiate	63 (78.8)	38 (90.5)	
Unresectable factor				0.348
	One	58 (72.5)	27 (64.3)	
	Multi-unresectable factors	22 (27.5)	15 (35.7)	
Clinical response				0.812
	CR/PR	42 (52.5)	23 (54.8)	
	SD/PD	38 (47.5)	19 (45.2)	
Residual tumor				0.943
	R0	74 (92.5)	39 (92.9)	
	R1/R2	6 (7.5)	3 (7.1)	
ypT stage AJCC 7th				0.46
	T0	2 (2.5)	3 (7.1)	
	T1	7 (8.8)	1 (2.4)	
	T2	9 (11.3)	7 (16.7)	
	T3	29 (36.3)	17 (40.5)	
	T4a	29 (36.3)	12 (28.6)	
	T4b	4 (5.0)	2 (4.8)	
ypN stage AJCC 7th				0.275
	N0	22 (27.5)	16 (38.1)	
	N1	12 (15.0)	5 (11.9)	
	N2	15 (18.8)	8 (19.0)	
	N3	31 (38.8)	13 (31.0)	
Pathological response (Mandard grade)				0.06
	TRG1	7 (8.8)	2 (4.8)	
	TRG2	23 (28.8)	6 (14.3)	
	TRG3	14 (17.5)	10 (23.8)	
	TRG4	26 (32.5)	15 (35.7)	
	TRG5	10 (12.5)	9 (21.4)	

Abbreviation: AJCC 7th, *the 7th American Joint Committee on Cancer (AJCC) TNM Staging Classification for Carcinoma of the Stomach*; TRG, tumor regression grade; CR, complete response; PR, partial response; SD, stable disease; PD, progressive disease.
